# Rapid Detection Strategies for the Global Threat of Zika Virus: Current State, New Hypotheses, and Limitations

**DOI:** 10.3389/fmicb.2016.01685

**Published:** 2016-10-24

**Authors:** Shruti Shukla, Sung-Yong Hong, Soo Hyun Chung, Myunghee Kim

**Affiliations:** ^1^Department of Food Science and Technology, Yeungnam UniversityGyeongsan-si, South Korea; ^2^School of Biosystem and Biomedical Science, College of Health Sciences, Korea UniversitySeoul, South Korea

**Keywords:** detection, diagnosis, immunoassay, liposome, PCR, Zika, virus

## Abstract

The current scenario regarding the widespread Zika virus (ZIKV) has resulted in numerous diagnostic studies, specifically in South America and in locations where there is frequent entry of travelers returning from ZIKV-affected areas, including pregnant women with or without clinical symptoms of ZIKV infection. The World Health Organization, WHO, announced that millions of cases of ZIKV are likely to occur in the USA in the near future. This situation has created an alarming public health emergency of international concern requiring the detection of this life-threatening viral candidate due to increased cases of newborn microcephaly associated with ZIKV infection. Hence, this review reports possible methods and strategies for the fast and reliable detection of ZIKV with particular emphasis on current updates, knowledge, and new hypotheses that might be helpful for medical professionals in poor and developing countries that urgently need to address this problem. In particular, we emphasize liposome-based biosensors. Although these biosensors are currently among the less popular tools for human disease detection, they have become useful tools for the screening and detection of pathogenic bacteria, fungi, and viruses because of their versatile advantageous features compared to other sensing devices. This review summarizes the currently available methods employed for the rapid detection of ZIKV and suggests an innovative approach involving the application of a liposome-based hypothesis for the development of new strategies for ZIKV detection and their use as effective biomedicinal tools.

## Overview: What Is Zika and How Did it Become Epidemic?

Zika virus (ZIKV), the causative agent of the infectious disease Zika fever, is a positive-sense RNA virus that belongs to the family *Flaviviridae*, genus *Flavivirus*, and is similar to Dengue virus (DENV), yellow fever virus, Japanese encephalitis virus, and West Nile virus (Sikka et al., 2016). ZIKV was first isolated from *Rhesus macaques* in Uganda in 1947. Previously, only sporadic cases of negligible concern associated with human ZIKV infection were reported (Hayes, 2009). Now, ZIKV infections have become epidemic throughout the world (Charrel et al., 2016).

In the north-eastern states of Brazil, the public health authorities recently confirmed autochthonous transmission of ZIKV with the first known reported case of ZIKV infection in mainland South America (Campos et al., 2015; Zanluca et al., 2015), followed by 26 countries, including countries in the European Union and the outermost regions of the Americas, such as Barbados, Bolivia, Brazil, Colombia, Costa Rica, Curacao, Dominican Republic, Ecuador, El Salvador, French Guiana, Guadeloupe, Guatemala, Guyana, Haiti, Honduras, Jamaica, Martinique, Mexico, Nicaragua, Panama, Paraguay, Puerto Rico, Saint Martin, Suriname, the US Virgin Island, and Venezuela (Pan American Health Organization [PAHO], 2016; World Health Organization [WHO], 2016). An increased frequency of ZIKV infection among world travelers has been reported in European countries, including Austria, Denmark, Finland, France, Germany, Ireland, Italy, Portugal, the Netherlands, Spain, Sweden, Switzerland, and the UK (European Centre for Disease Prevention [ECDC], 2016).

The virion of ZIKV consists of an approximately 11 kb positive-sense RNA with a single capsid and two membrane-associated envelope proteins (M and E) (Leyssen et al., 2000; Daep et al., 2014; Charrel et al., 2016). Recent outbreaks of ZIKV infections have become fatal on a daily basis in the Americas, where this obscure viral candidate has been placed at the forefront of global healthcare. The reported occurrences of ZIKV infections are thought to be transmitted mainly by the mosquito species *Aedes aegypti* and *Aedes albopictus*. Infections have now dramatically increased in highly populated areas of South, Central, and North America due to the increased frequency of the international travel from Zika-infected areas (Bogoch et al., 2016). Considering the calamity of ZIKV infection, there is an urgent need to develop rapid detection methods for ZIKV along with DENV, which shares common clinical symptoms with ZIKV. The purpose of this review is to provide a complete update of the various analytical methods for virus detection, such as molecular, immunological, sensor-based and other detection assays, along with the advantages and limitations of these strategies. Furthermore, we suggest innovative hypothetical approaches for the development of liposome-based rapid detection assays for ZIKV detection, which will provide new insight to medical professionals for controlling this widespread epidemic virus candidate.

## Diagnostic Features and Challenges

ZIKV, an emerging flavivirus, shares common clinical symptoms with DENV and chikungunya virus (CHIKV). The outbreaks caused by these viruses present a large number of diagnostic challenges. The clinical manifestations of ZIKV involve similar clinical symptoms to DENV and CHIKV, which include fever, exanthema, conjunctivitis, retro-orbital headache, and arthralgia (Cardoso et al., 2015). The diagnosis of viral infection has specific management implications for medical personnel. The identification of DENV requires a routine follow-up to examine thrombocytes along with hematocrit, whereas for CHIKV, chronic arthralgia should be assessed due to its high prevalence. In the case of ZIKV, a detailed diagnosis of sexual and maternal-fetal transmission should be performed to confirm the risk of congenital microcephaly in newborn babies (Fauci and Morens, 2016). A variety of arboviral infections (arthropod-borne; DENV is the most common arboviral infection) may have similar clinical presentations; therefore, their circulation may be under-reported if specific diagnostic tools have not been implemented. However, there are several drawbacks in ZIKV diagnosis due to the lack of availability of diagnostic tools and the frequent cross-reactivity of antibodies between flaviviruses, which have resulted in several limitations in the use of serology (Musso et al., 2015). Commonly, no routine testing of virus cultures is performed, and an antigenic detection test is lacking at present (Musso et al., 2015; Saiz et al., 2016).

The symptoms of ZIKV infection usually tend to be mild, and the initial symptoms can escape notice, reducing the opportunity to collect a sample. Although the viremic period has not been completely defined, viral RNA has been detected in serum after the onset of symptoms up to day 10. In addition, RNA particles of ZIKV have been detected in urine over an extended period in the acute phase, leading to the possibility of considering an alternative sample type. Evidence suggests that serum samples should be taken during the first 5 days after the onset of symptoms supported in some more detailed studies (Musso et al., 2015). Symptoms of microcephaly associated with ZIKV during the development of newborns in the uterus have been reported (Oduyebo et al., 2016). For the diagnosis of infant microcephaly, a complete analysis of head circumference is requested (Kallen, 2014), as the diagnostic parameters for severe microcephaly include a head circumference more than 3 standard deviations below the mean (Von der Hagen et al., 2014). Testing should be performed in pregnant women with positive or inconclusive results from ZIKV testing. If diagnostic parameters confirm possibility of congenital ZIKV infection in an infant, further clinical evaluation should be performed in follow-up. Fever is a common presenting symptom in patients testing positive for arboviruses due to their association with multiple illnesses; hence, it is suggested to eliminate differential diagnoses (Kelser, 2016). Patients with DENV and ZIKV present with temperatures >40°C and <38.5°C, respectively. ZIKV is usually self-limiting, with symptoms lasting 2 to 7 days. Jaundice is a distinguishing clinical presentation of yellow fever virus and can aid in identifying patients with ZIKV virus. The presence of nausea, vomiting, and bleeding may be helpful in identifying DENV. Any of the above symptoms in an individual who has been exposed to ZIKV indicates the possibility of ZIKV infection, and immediate serum testing should therefore be performed (Centers for Disease Control and Prevention [CDC], 2015a,b).

## Available Detection Methods for ZIKV

**FIGURE 1:**
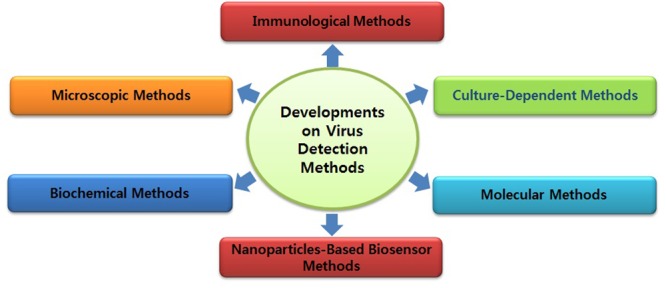
**Developments in various pathogen detection strategies**.

**FIGURE 2:**
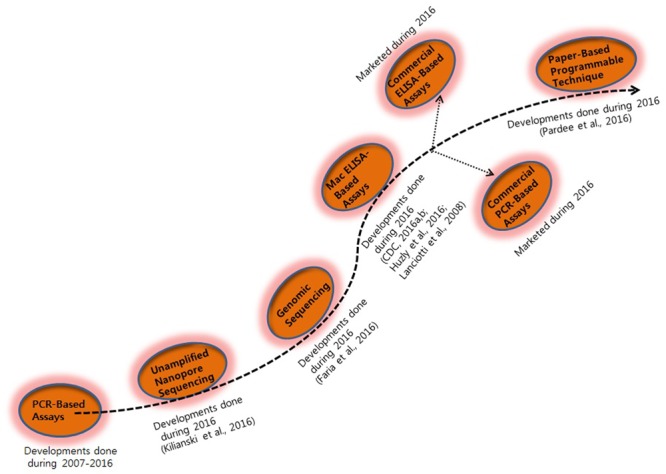
**Consistent major year-wise efforts in the development of detection assays for Zika virus**.

Diagnosis of ZIKV in the laboratory is usually based on the serum analysis employing viral RNA or antibody-based detection assays (Al-Qahtani et al., 2016; Haug et al., 2016). However, molecular technique-based assays are considered more reliable due to the cross-reactivity of IgM antibodies among the candidate flaviviruses during serological analysis (Haug et al., 2016). Consistent efforts are being made regarding the development of various detection strategies for virus detection (**Figures 1** and **2**). However, there is still a need to search for and improve upon more sensitive and reliable detection methods for the challenging virus candidate ZIKV for daily diagnoses. Developed and applied methods for the detection of ZIKV are listed in **Table 1**.

**Table 1:** Methods available and applied for Zika virus detection.

Method	Sample analyzed	Virus strain	Detection limit	Reference
Real-time reverse transcription-PCR	Serum specimens	Flavivirus/Zika virus (ZIKV)	Tested for positive/negative samples	Lanciotti et al., 2008
Real-time reverse transcription-PCR	Serum specimens	Flavivirus/ZIKV	Tested for positive/negative samples	Lanciotti and Tesh, 2011
One step real-time reverse transcription-PCR	Human serum	ZIKV	0.05 plaque forming unit (pfu) in less than 3 h	Faye et al., 2013
Viral metagenomic next-generation sequencing	Amniotic fluid samples	ZIKV	Tested for positive/negative samples	Calvet et al., 2016
Complementary DNA synthesis followed by PCR	Human serum	ZIKV	33.7 pfu/mL	Faye et al., 2008
Recombinant polymerase amplification assay	Virus culture	Dengue virus (DENV)	Analytical sensitivity between 10^5^ and 10^3^ RNA molecules	Wahed et al., 2015
Real-time-PCR	Virus cell culture/Human serum	Flavivirus/ZIKV	140 copies viral RNA/reaction	Balm et al., 2012
Multiplex real-time reverse transcription-PCR	Virus cell culture/Human serum	Flavivirus/ZIKV/DENV/ Chikungunya virus	10^8^ to 10 copies/μL for ZIKV and from 10^8^ to 10^2^ copies/μL for Dengue-1	Waggoner et al., 2016
Real-time-PCR	Saliva/Blood	ZIKV	Tested for positive/negative samples	Musso et al., 2015
Genomic sequencing	Serum/Blood/Amniotic fluid/ New born babies	ZIKV	Tested for positive/negative samples	Faria et al., 2016
Paper-based synthetic gene networks	Virus cell culture (ZIKV RNA genome)/Plasma samples	ZIKV	1.7 × 10^6^ copies/mL	Pardee et al., 2016
Zika-specific reverse transcriptase-PCR	Human serum	ZIKV	No information available	Charrel et al., 2016
Zika MAC-ELISA	Virus cell culture/Human serum	ZIKV	No information available	Centers for Disease Control and Prevention [CDC], 2016a,b; Lanciotti et al., 2008
Indirect immunofluorescent assay	Human serum	ZIKV	Tested for positive/negative samples	Tappe et al., 2014
Antigen detection and immunoglobulin M capture ELISA	Virus cell culture/Human serum	Mosquitoes transmitted virus/Yellow fever virus	1.0 × 10^3^ pfu/100 mL	Adungo et al., 2016
Real time-PCR	Human urine	ZIKV RNA	Tested for positive/negative test	Gourinat et al., 2015
Instrument-free point-of-care molecular detection (reverse-transcription loop-mediated, isothermal amplification assay)	Virus cell culture	ZIKV	5 pfu	Song et al., 2016
Streptavidin-magnetic nanoparticles coupled oligonucleotide detection based on loop-mediated isothermal amplification	Virus cell culture/Human serum	ZIKV	1 aM synthetic ZIKV oligonucleotide	Tian et al., 2016
Liposome-based detection assay	Virus cell culture	DENV	10 pfu/mL	Baeumner et al., 2002
Multi-analyte single-membrane biosensor	Virus cell culture/Human serum	DENV	50 RNA molecules for serotype 2, 500 RNA molecules for serotypes 3 and 4, and 50,000 RNA molecules for serotype 1	Zaytseva et al., 2004

### Molecular Methods

Molecular detection of viruses is a two-step process that includes virus detection in propagated cell culture and viral genome-based detection through molecular amplification using polymerase chain reaction (PCR)- or real-time PCR-based detection methods. In propagated cells, the detection of a virus is confirmed by its cytopathic effect in cell culture, and positive results are confirmed using tissue culture with an infectious dose 50 (TCID_50_) in plaque assays (Hamza et al., 2011). During the last several years, there has been a revolution in virus detection methods using real-time PCR based assays, which can be performed rapidly and produce specific, sensitive, and reproducible results for virus detection at a very low concentration (Sanchez et al., 2007). All of these parameters are interconnected and depend mostly on the target sequences of primers and probes and provide absolute specificity and a balance between high sensitivity, broad reactivity, and reliability of quantification. (Seifi et al., 2012).

Polymerase chain reaction-based methods have numerous advantages compared with conventional assay methods, such as rapidity, quantitative measurement, low contamination rates, and easy standardization (Sanchez et al., 2007; Seifi et al., 2012). Although a real-time reverse transcription PCR assay is available for the detection of Micronesian ZIKV strains in Africa and Asia, it does not cover the genetic diversity and geographic distribution of ZIKV (Hayes, 2009). Current diagnostic techniques for ZIKV infection are time-consuming and are mainly based on specific detection of antibodies or isolation of viruses from animals or mosquitoes (Digoutte et al., 1992; Hayes, 2009; Faye et al., 2013). However, standard PCR-based techniques such as real-time PCR and quantitative real-time PCR provide a rapid, specific, and sensitive method for early detection of ZIKV (Foy et al., 2014). Faye et al. (2013) developed a quantitative real-time PCR assay for the detection of ZIKV in mosquitoes in the field with high analytical sensitivity. It was found that the assay was able to detect 37 ZIKV isolates. These findings confirmed that the use of real-time reverse transcription PCR assays could be a useful tool for the detection of ZIKV in virus pandemic areas where viruses including DENV and CHIKV also occur (Faye et al., 2013).

Calvet et al. (2016) developed a real-time quantitative PCR assay for the detection of ZIKV in pregnant women using viral metagenomics and gene sequencing analysis. The real-time quantitative PCR analysis confirmed that the developed assay was able to detect ZIKV infection in amniotic fluid; however, the same assay showed negative results for the detection of ZIKV infection in urine and serum samples. Escadafal et al. (2014) tested the efficacy of a recombinant polymerase amplification assay for the detection of yellow fever virus using a nucleic acid detection method, which confirmed positive results. Detection of DENV was also confirmed using a recombinase polymerase amplification assay, supporting the suggestion that this assay could be a useful tool for the detection of ZIKV (Wahed et al., 2015). Due to the rapid global increase in ZIKV and concern about the rapid increase of microcephaly cases in newborns, the development of specific and sensitive point-of-care-testing methods has become a major public health priority (Sikka et al., 2016). Because ZIKV shares a common platform of clinical manifestation with DENV and CHIKV, a specific and sensitive one-step real-time PCR assay was tested for the presence of ZIKV in 88 Dengue and Chikungunya-negative sera samples collected from the patients representing with DENV-like illness in Singapore (Balm et al., 2012). The assay displayed specific detection of ZIKV with a low detection limit (140 copies of viral RNA), suggesting that this assay could be useful for viral detection in a variety of environmental samples (Balm et al., 2012). Recently, Waggoner et al. (2016) developed and evaluated a multiplex real-time reverse transcription PCR assay for simultaneous detection of ZIKV. The developed assay improved the detection limit of ZIKV relative to the corresponding real-time reverse transcription PCR assay, and 31 samples were found to be positive for ZIKV (Waggoner et al., 2016). The findings of this study suggested that improved sensitivity for ZIKV detection is needed, given the low viremia detected in clinical samples and the current lack of accurate alternative diagnostics, such as serology (Lanciotti et al., 2008; Musso et al., 2015). Additionally, the developed assay identified 17 co-infections in ZIKV-positive patients, suggesting its potential for application as a multiplex diagnostic test for viral detection.

Furthermore, Kilianski et al. (2016) developed an unamplified RNA/complementary DNA (cDNA)-hybrid nanopore sequencing assay for the detection of RNA viruses, including Middle East respiratory syndrome, Venezuelan equine encephalitis virus, Ebola, and ZIKV. The developed method was able to detect Venezuelan equine encephalitis virus within 3 hours of acquisition and achieved differentiation from other viral genomes, facilitating strain-level identification. Nanopore sequencing is a novel genomics technology, which has potential applications for routine biosurveillance, clinical diagnosis, and outbreak investigation of virus infections by rapid sequencing of unamplified RNA/cDNA hybrids. Kilianski et al. (2016) sequenced unamplified poly(A)-tailed viral RNA using a rapid cDNA library preparation coupled with real-time data analysis to determine its potential application for pathogen genomic characterization. This approach for pathogen identification and characterization differs from the previously used methods on the MinION platform (Hoenen et al., 2016). Biased techniques, such as amplicon sequencing, have proven to be effective in complex sample backgrounds in which titers of the target pathogen might be low, but such approaches limit characterization to known pathogens and require additional viral genome amplification (Wang et al., 2015; Quick et al., 2016). The great potential of the use of a RNA/cDNA hybrid approach in field studies has been confirmed in western Africa (Hoenen et al., 2016; Quick et al., 2016). Generally, high virus titers in clinical samples are necessary for virus detection before genome sequencing, hence utilizing an RNA/cDNA hybrid approach for genomic ZIKV characterization could be a feasible strategy, especially for genomic library preparation and for reducing the time required for strain-level identification (Faria et al., 2016).

Although PCR-based molecular techniques have aided in the easy detection of a variety of viruses, there are still several limitations to these methods. It has been noted that these methods are prone to miss detections, favoring false negative results and requiring proper quality control measures. However, precautions have been taken to overcome the problem of inhibition, including sample dilution analysis, the use of a small sample size, and adaptation of the PCR assay employing chemical reagents such as Tween (Al-Soud and Radstrom, 2001; Rutjes et al., 2005; Butot et al., 2007). Another limitation of PCR-based methods is their inability to differentiate between infectious and non-infectious viruses. Although several approaches have been used to overcome this limitation, the utilization of integrated systems based on the molecular detection of viruses after cell culture infection is considered one of the most efficient methodologies (Reynolds et al., 2001; Rodriguez et al., 2009). Additionally, integrated cell culture-PCR assays allow rapid virus detection with selective enumeration of infectious viruses, though the long incubation period required to reveal a cytopathic effect is a major limitation.

### Paper-Based Synthetic Gene Networks for ZIKV Detection

The aim of synthetic biology focuses on re-engineering of the molecular components of cells to exploit the power of biology for which molecular biologists have developed whole-cell biosensors, synthetic probiotic candidates, new drug sources, green technology, and chemical resources (Zhang et al., 2012; Torella et al., 2013; Fossati et al., 2014; Kotula et al., 2014). As recently reported, paper disk diagnostic tools show significant efficacy as rapid and low-cost screening tools for the screening of blood, urine, and saliva samples for specific identification of virus strains. The assay methodology is based on the color change of a paper disk to purple, indicating the presence of virus, or yellow, in the absence of virus. Recently, Pardee et al. (2016) reported some biotechnological parameters that dramatically lower costs and technical barriers in the development of synthetic biology-based diagnostics. In this regard, programmable RNA sensors referred to as “toehold switches” could be the first technology to be rationally applied to bind virtually any sense RNA sequence (Green et al., 2014). Second, a freeze-dried, paper-based, cell-free protein expression platform allows the application of these toehold switch sensors outside of laboratories by providing an abiotic, sterile method for the distribution and storage of genetic circuits at room temperature (Pardee et al., 2014). Concerning the above findings, combination of these techniques could provide a rapid and inexpensive platform for the development of easy-to-use virus diagnostic sensors. In the context of the ZIKV outbreak, paper-based sensors offer a solution to the critical challenges involved in diagnosis of the virus. Standard serological methodologies involving antibody detection present limitations due to their cross-reactivity with other flaviviruses detected in previously infected patients; thus, nucleic acid-based and isothermal nucleic acid amplification detection methods are recommended for accurate detection of viruses (Lanciotti et al., 2008; Tappe et al., 2014; Zammarchi et al., 2015). However, such techniques are relatively expensive, require technical expertise to run and interpret, and involve equipment that is incompatible with use in remote and low-resource locations, where surveillance and containment are critically needed.

### Immunoassays

The current ZIKV outbreak represents a great threat specifically for pregnant women. Because the antibody response during the pregnancy stage may differ from that in non-pregnant individuals, more precautions should be taken when determining the ZIKV immune responses in pregnant women. In flavivirus infections, immunoglobulin M (IgM) antibodies typically develop within a few days after the onset of illness and can generally be detected up to three months (Charrel et al., 2016). In addition, immunoglobulin G (IgG) antibodies are developed within a few days after development of IgM antibodies and can be detected for several months.

Recently (February 26, 2016), the Centers for Disease Control and Prevention (CDC) requested that a letter be issued authorizing the emergency use of the Zika IgM antibody capture enzyme-linked immunosorbent assay (Zika MAC-ELISA) for the presumptive detection of ZIKV-specific IgM in human sera or cerebrospinal fluid (Centers for Disease Control and Prevention [CDC], 2016b). The Zika MAC-ELISA is employed for *in vitro* qualitative detection of ZIKV-specific IgM antibodies in human sera. In addition, testing of ZIKV has been applied based on CDC clinical and epidemiological criteria for ZIKV which include clinical signs and symptoms associated with ZIKV infection and/or history of residence in or travel to a geographic region with active ZIKV transmission at the time of travel or other epidemiologic criteria that may indicate ZIKV testing (Centers for Disease Control and Prevention [CDC], 2016a). As reported previously, immune responses under ZIKV infection have only been described in a small number of patients (*n* = 11), during the ZIKV virus outbreak in Yap (Lanciotti et al., 2008). When MAC-ELISA for IgM and capture ELISA for IgG with the whole viral antigen (inactivated virus) and monoclonal antibodies (MAbs) were applied, IgM was found to appear as soon as 3 days after the onset of symptoms, while IgG appeared after 10 days in a patient with no history of previous flavivirus infections (Johnson et al., 2000; Martin et al., 2000). Subsequently, it became possible to detect neutralizing antibodies against ZIKV as early as 5 days after the onset of fever. Euroimmun (2016) has developed the first complete test package for the serological detection of ZIKV infections. The ELISAs and indirect immunofluorescence assays allow the determination of specific antibodies (IgM, IgG) against a variety of viruses in the blood of infected patients.

Huzly et al. (2016) reported that the use of putative cross reacting sera in ELISA tests from patients with Euroimmun anti-ZIKV IgG and IgM antibodies against ZIKV showed high specificity, confirming the applicability of Euroimmun ELISA for specific detection of virus in patients exposed previously to flavivirus or vaccine. The results suggest that this ELISA method could be an effective diagnostic tool for the screening and counseling of patients with a possibility of ZIKV virus infection, especially pregnant women and travelers commuting from ZIKV-endemic regions (Huzly et al., 2016). To confirm the ability of the ZIKV ELISA to detect ZIKV antibodies, Tappe et al. (2014) analyzed serum samples collected from 10 patients in Brazil suffering from acute ZIKV infection. Laboratory results confirmed that the indirect immunofluorescent assay was able to define immunofluorescent assay titers for anti-ZIKV IgM (1: 1,280 to 1: > 20,480) and anti-ZIKV IgG (1: 320 to 1: > 20,480) (Tappe et al., 2014). These samples were previously confirmed to be negative for IgM and IgG against DENV and for the DENV nonstructural protein-1(DENV NS1) antigen.

Along with ZIKV and DENV, yellow fever is another acute viral infection transmitted through mosquito bites. It is strongly believed that the current emergence and re-emergence of yellow fever and other arboviruses is partly attributed to the increased migration of people from disease-endemic regions and the expanding establishment of the vector. The use of flavivirus antigens in the development of MAbs and diagnostic tests has not been well documented (Vazquez et al., 2009; Nunes et al., 2011; Okamoto et al., 2012; Kwallah et al., 2013; Escadafal et al., 2014). The application of MAbs in immunoassays offers the advantage of high specificity, due to their ability to bind to specific epitopes of antigens. This specific binding is useful in reducing the incidence of cross-reactivity, which is reported to negatively impact the use of many serological kits (Modis et al., 2003, 2005). Adungo et al. (2016) also reported the development, characterization, and evaluation of MAbs to yellow fever virus. Diagnostic application of these MAbs was tested in antigen detection and IgM capture ELISA. The results suggested that MAb-based antigen detection ELISA enabled the detection of virus in 40 culture supernatants containing titers of approximately 1,000 plaque forming units (pfu). Concerning the above findings, the developed approach can be applied for the development of improved detection kits for ZIKV. One of the limitations of this test is the possibility of false positive results in patients with a history of infection with other flaviviruses. Additional testing of equivocal and positive specimens and/or other patient-matched specimens, as specified in the CDC-issued algorithm, is therefore required to confirm ZIKV infection (Centers for Disease Control and Prevention [CDC], 2016a).

### Magnetic Nanoparticles-Based Assays

Zika fever is a zoonotic infection caused by ZIKV, which shares the limelight with other well-known members of the flavivirus family, such as DENV, yellow fever virus, West Nile virus, and Japanese encephalitis virus (Tilak et al., 2016). Consequently, there is an urgent need to develop effective methods for the surveillance of ZIKV. As ZIKV and DENV share sequential homology, there is a high possibility that there may be detection principles and vaccine development strategies that are applicable to both viruses with some modifications.

One approach that could be useful for the detection of DENV involves concentrating virus particles using ultracentrifugation and polyethylene glycol-mediated precipitation. Although these methods have been used successfully for variety of viruses, ultracentrifugation shows numerous practical limitations, such as the great amount of time required, which may increase the false-positive rate when applied for PCR analysis (Roth et al., 1999). Regarding polyethylene glycol-mediated precipitation, although it is simple and easy to perform, it interferes with subsequent PCR analysis (Novotny et al., 1992). A possible alternative to these methods could be application of magnetic beads coated with molecules that efficiently bind to the virus. These beads allow viral particles to be captured and assist in the concentration of viral particles through the application of magnetic field.

A number of magnetic nanoparticles, such as iron, nickel, or cobalt, have been successfully used for biomedical and environmental applications due to their highly/specific surface area and the ease of the magnetic collection of target materials adsorbed by the magnetic nanoparticles (Safarikova and Safarik, 2001; Pankhurst et al., 2003). However, these beads generally exhibit inherent chemical instability, limiting their application in the fields of biological and environmental science (Saraswati et al., 2012). To overcome this limitation, magnetic nanoparticles have typically been encapsulated with a protective shell of graphite, silica, or polymer for chemical stability (Saraswati et al., 2012). Similarly, Sakudo et al. (2016) developed a novel technology for the sensitive detection of DENV using graphite-encapsulated magnetic nanoparticles conjugated with anti-DENV antibody. Their assay method demonstrated that antibody-integrated magnetic beads were useful for capturing DENVs. The capturing of DENV1-4 using antibody-integrated magnetic beads was confirmed by the results of the real-time PCR analysis, showing that the captured fraction contained DENV genomic RNA (Sakudo et al., 2016). Therefore, this method may be used in combination with real-time PCR for the detection of DENV and may increase the sensitivity of viral detection for the diagnosis of DENV. Based on these findings, it can be hypothesized that this methodology could be extended for the accurate detection of ZIKV through the immobilization of an anti-ZIKV antibody on the functionalized surface of graphite magnetic nanoparticles. The modified graphite magnetic nanoparticles can then be assessed for their ability to capture ZIKV, and the concentrated virus can subsequently be detected through the application of PCR-based amplification procedure.

In the case of hydrophobic graphite-encapsulated magnetic nanoparticles, which present limitations in various biomedical applications, the particles can be modified appropriately to achieve improved binding properties, allowing them to efficiently recognize and bind to molecular targets, including antibodies, antigens, and receptors (Poplawska et al., 2014). Improvement of amino group functionalization can increase the reactivity and hydrophobic nature of graphite particles with the desirable functionality of graphite (Saraswati et al., 2012). A promising method for the amino functionalization of magnetic graphite nanoparticles could be to use inductively coupled radiofrequency plasma, an environmentally friendly method that requires a short duration for the reaction to reach completion, thus effectively introducing amino groups (Saraswati et al., 2011). Furthermore, alteration of plasma discharge conditions can help to optimize the degree of surface derivatization with the amino groups of graphite magnetic nanoparticles. With these modifications of graphite magnetic nanoparticles, efficient surface immobilization of antibodies against various pathogens, including anti-influenza virus and anti-*Salmonella* antibodies, could be achieved (Sakudo et al., 2015).

Recently, Tian et al. (2016) developed an attomolar ZIKV oligonucleotide detection method based on loop-mediated isothermal amplification and susceptometry. It was demonstrated that hydrodynamic volumes of streptavidin-magnetic nanoparticles were dramatically increased after a successful loop-mediated isothermal amplification reaction. The hydrodynamic volumes are probed as Brownian relaxation frequency shifts, which can be employed to quantify the ZIKV oligonucleotide. The proposed detection system can recognize 1 aM synthetic ZIKV oligonucleotide in 20% serum with a total assay time of 27 min. The results suggested that this could be a promising strategy that may give rise to new possibilities for diagnosing and controlling ZIKV infection.

With the increasing incidence of Dengue infection in developing countries where DENV is endemic, the rapid applicability of diagnostic tests is required for disease control. Several immunological approaches have been applied for laboratory diagnosis of DENV infection. These methods include detection of the virus (by cell culture and immunofluorescence-based), detection of virus antigen (by ELISA), detection of anti-DENV antibody (by hemagglutination inhibition, complement fixation test, and neutralization tests), and detection of virus nucleic acid (by real-time reverse transcription-PCR). The hemagglutination inhibition is the most widely accepted serological technique in developing countries; however, ELISA has become the technique that is most often applied for the serological diagnosis of DENV infection in DENV epidemic regions due to its stability, simple handling procedure, and no need of any complicated equipments.

### Developments in the Liposome-Based Detection of ZIKV/DENV

The characterization and detection of viruses is a labor-intensive and time-consuming process. Upon the emergence of new viral pathogens with a high frequency and catastrophic outbreaks, there is always a need for improved detection and identification methods to control the proliferation of these pandemic viral candidates. Currently, at laboratory scale, only PCR-based molecular techniques provide accurate detection of a variety of viruses based on trial-and-error methods using a cell culture-based procedure. PCR-based methods require thermal cycling instruments and transformation of DENV/ZIKV genomic RNA to DNA (Kow et al., 2001; Zaytseva et al., 2004). Zaytseva et al. (2004) developed a multi-analyte single-membrane biosensor for the serotype-specific detection of DENV, which showed 92% reliability in DENV serotype determination. The assay exhibited a detection limit of 50 RNA molecules for serotype 2; 500 RNA molecules for serotypes 3 and 4; and 50,000 RNA molecules for serotype 1, following isothermal amplification of the target sequences. The developed biosensor can be considered as an applicable portable, inexpensive, and easy to use tool, representing an alternative to current detection methods based on more expensive and time-consuming methods, such as ELISA or tissue culture.

Although these techniques are readily available, they are expensive and present engineering challenges regarding miniaturization and field utilization. In addition, PCR products, which consist of double-stranded DNA, must be denatured before being subjected to a probe hybridization-based detection method (Baeumner et al., 2002). In contrast, biosensors based on liposome technology have been successfully used for the development of rapid, inexpensive, and field-utilizable detection systems with potential for the detection and quantification of RNA molecules (Esch et al., 2001). We provide an update on the development of liposome-based detection methods for DENV or ZIKV, as the two viruses share majority of their genetic information. Liposome-based immunoassays show applicability in various areas of food chemistry, food microbiology, nano-biotechnology and diagnostic or clinical microbiology, for the detection of foodborne pathogens, toxins, and hazardous components in a variety of environmental and human samples. Liposome immunoassays involve a liposome-encapsulating marker, prepared with a phospholipid composition and coupled to either an analyte or antibody through a suitable procedure (Shukla et al., 2012). There have been various recent advances in methods related to liposome immunoassays, including immunoliposome (liposome coupled to antibody)-based ELISA, immunoliposome-based magnetic separation, and liposome-based immunochromatographic strips used in various pathogen detection applications. Baeumner et al. (2002) developed a liposome biosensor-based method for the detection of DENV that was sensitive, rapid, and serotype specific. The biosensor assay was based on utilizing a membrane-based DNA/RNA hybridization system with liposome amplification. The generic DNA probe was coupled to dye-encapsulating liposomes, and conserved or DENV serotype-specific probes were immobilized on a polyether sulfone membrane strip. Liposomes were mixed with the amplified target sequence and then applied to the membrane. The mixture was allowed to migrate along the test strip, and the liposome target sequence complexes were immobilized in the capture zone via hybridization of the capture probe with the target sequence. The amount of liposomes present in the immobilized complex is directly proportional to the amount of target sequence present in the sample and can be quantified using a portable reflectometer (Baeumner et al., 2002). Connelly et al. (2012) also developed a method involving microfluidic pre-concentration coupled to liposome-based signal amplification to efficiently detect viruses in environmental water and clinical samples. Subsequently, an electrochemical liposome-based assay was developed to facilitate direct detection of virus-liposome-bead complexes using a magnet (Connelly et al., 2012). The integrated current signals from the lysis of captured liposomes resulted in detection of the viral titer in an environmental sample. Although a number of established liposome-based methods are available for the detection of different viruses in various samples, no specific and sensitive liposome-based detection method has been developed for ZIKV.

### Liposome-Based Detection Assays in Medical/Clinical Use

A number of liposome-based assays are also being used in biomedical and therapeutic applications as clinical diagnostic assays. For pathogen detection, liposome-based lateral flow assays coupled with nucleic acid sequence-based amplification have been proved to represent an effective alternative for the rapid and sensitive detection of viable bacterial pathogens (Hartlefy and Baeumner, 2003; Baeumner et al., 2004). Edwards et al. (2010) developed a fluorescent dye-encapsulated liposome tagged with DNA aptamers and used it for the detection of alpha-thrombin via an aptamer-based assay in human plasma. The advantages of DNA aptamers over antibodies include stability; production via inexpensive, consistent chemical synthesis; ease of modification in terms of both labeling and selective changes in the sequence to achieve affinity selectivity and enhanced stability; potential for regeneration; smaller size; and production against toxic or poorly immunogenic targets (Tombelli et al., 2005; Balamurugan et al., 2008). In these assays, dye-encapsulating liposomes are used for signal enhancement, providing extremely low detection limits in the assays. In addition to fluorescent markers and enzymes, liposomes can be used to encapsulate a variety of electrochemical markers. Feng et al. (2010) and others (Sofou and Sgouros, 2008; Sajja et al., 2009) developed and demonstrated the utility of a bifunctional immunoliposome system targeting glioma cells both *in vitro* and *in vivo*, providing the potential for drug delivery and imaging in tumors. Lehtinen et al. (2012) also reported an immuno-targeting system involving liposomal drug carriers to treat ovarian carcinoma. Additionally, immunoliposomes have been used to deliver contrast agents and radionuclides for diagnostic imaging and therapy (Torchilin, 2000; Erdogan et al., 2008). Semiconductor quantum dots conjugated to liposomes are used for imaging and the immunoliposomes have successfully been observed *in vitro* and *in vivo* (Weng et al., 2008).

Under this subtitle, we intended to elucidate the variety of ways in which liposomes have been used as analytical reagents to date. These methods relying on a tagged liposome could provide numerous ways for signaling molecules to be released to provide a signal. Therefore, it can be stated that these liposomes/immunoliposomes show potential for application in various industrial and medical sectors, including for virus detection through liposome immunoassays.

## Commercially Available Test Kits for ZIKV Detection

As a future consideration, commercial serology tests will become increasingly available for the detection of a variety of viruses. To our knowledge, there are few commercial tests that are expected to hit the commercial market in very near future. ZIKV IgG or IgM ELISA kits based on utilizing a double-antigen sandwich ELISA will be introduced by MyBiosource in USA. However, no information on the type of antigens involved or the specifics of validation for determining cross-reactivity is available. Biocon Diagnostics in Canada offers a rapid finger-prick assay based on a mixture of ZIKV NS1 and envelope protein that can detect IgM and IgG viral antibodies. The company indicates a specificity of 99%, but no specifications regarding the validation procedure are given. Euroimmun in Germany offers an IgM/IgG immunofluorescence assay and an IgM/IgG ELISA based on a NS1 protein. The ZIKV immunofluorescence assay is offered in a mosaic slide, together with assays for DENV1-4 and CHIKV. The provided information indicates cross-reactivity with antibodies directed against tick-borne encephalitis virus, West Nile virus, and DENV for both the IgG and IgM assays (Charrel et al., 2016). Furthermore, validation data indicate a wide range of specificities and sensitivities for the IgM and IgG ZIKV immunofluorescence assays depending on the validation cohort. However, the provided values are difficult to interpret as the description of the cohorts is too brief. In addition, these data should be interpreted with great caution as positivity was only rated in the cut-off dilution. This could mean that the specificity can be different than given (higher) as the results were not scored as end-titers. The use of end-titers would provide a window for differentiating the cross-reactivity measured. The Euroimmun ZIKV ELISA is based on recombinant NS1 protein which leads to a reduction of cross-reactivity with other flavivirus antibodies to maximal values of 18.8% (IgG) and 8.3% (IgM). Euroimmun appears to be the only manufacturer that is actually providing detailed validation data, and its data clearly address and illustrate the above-mentioned difficulties with cross-reactivity in flavivirus sero-diagnostics (Euroimmun, 2016).

Musso et al. (2015) performed a molecular detection of ZIKV in saliva samples after RNA extraction using NucliSENS^®^ easyMAG^®^ System (BioMérieux) according to manufacturer’s recommendations. Extracted RNA was used for further real-time PCR detection system using two real-time primers/probe amplification sets specific for ZIKV (Musso et al., 2015). The analysis confirmed 210 positive results from 748 blood samples (28.1%) and 182 positive results from 319 saliva samples (57.1%) as the overall number of real-time PCR-positive ZIKV samples, suggesting that ZIKV RNA was more frequently detected in saliva samples than blood samples (Musso et al., 2015). Updated information on newly developed commercial detection kits for ZIKV detection is given in **Table 2**.

**Table 2:** Update on commercially available detection kits for Zika virus.

Commercially available detection kits	Company
Immunoglobulin G (IgG) and immunoglobulin M (IgM) ELISA kit for Zika virus (ZIKV)	MyBiosource (USA)
Rapid finger prick assay kit (based on ZIKV nonstructural protein-1 and envelope protein)	Biocon Diagnostics (Canada)
IgM and IgG immunofluorescence kit for ZIKV	Euroimmun Diagnostics (Germany)
ZIKV IgM ELISA, Mac ELISA kit for ZIKV	InBIOS Diagnostics (USA)
Real star ZIKV real-time polymerase chain reaction kit	Altona Diagnostics (Germany)
Immunochromatographic test strips for ZIKV	Tanaka Diagnostics (Japan)
Fast-Track diagnostics ZIKV test kit based on- multiplex real-time polymerase chain reaction by TaqMan technique	Fast-Track Diagnostics (Malta)
ZIKV/DENV test kit based on RNA, qualitative real-time Reverse Transcriptase Polymerase Chain Reaction and ZIKV antibody (IgM), MAC-ELISA	Quest Diagnostics (USA)

## Recent Research Based on a Specific Hypothesis

The limitations of current diagnostic tests for ZIKV have been described previously. These considerations highlight technical challenges in test development and the limits of our scientific understanding of the virus at this stage of the ZIKV response. In our previous work, we reported polyclonal antibody production, purification, and applicability in various liposome-based assays (Shukla et al., 2012, 2016). There are currently no available studies on liposome-based assays for the detection of ZIKV, and only few such studies on DENV are available, which were based on liposomes. Considering the various assays developed for DENV and other viruses, we propose a strategy for developing a liposome-based assay for the detection of ZIKV. Thus, a specific liposome-based assay can be applied with our developed liposome particles to develop a liposome-based detection system for analyzing ZIKV in clinical samples using anti-ZIKV antibody.

In several reports, we have described the principle of various liposome-based assays for detecting pathogens (Shin and Kim, 2008; Shukla et al., 2012, 2016). Liposome immunoassays involve a liposome-encapsulating marker (sulforhodamine B dye) and are prepared with a phospholipid composition, coupled to either an analyte or antibody through a suitable procedure (Shukla et al., 2012, 2016), after which the assay is carried out in a routine manner. The detectable signal of these assays is produced upon the lysis of the liposome and the release of the encapsulated markers. Shin and Kim (2008) reported that as the size of the liposomes increased, a higher fluorescence signal was obtained due to a greater number of sulforhodamine B molecules encapsulated in the liposome. Various advanced methods related to immunoliposome assays have recently been developed, including immunoliposome-coupled ELISA, immunoliposome-coupled magnetic separation, and immunoliposome-based immunochromatographic strips used in various applications.

**FIGURE 3:**
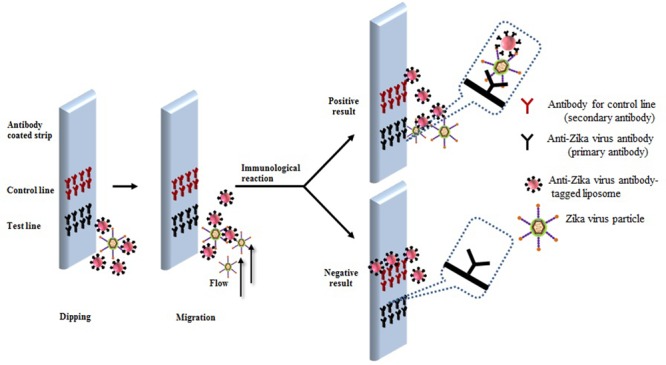
**Research theme for developing liposome-based immunochromatographic strips for specific Zika virus**.

**FIGURE 4:**
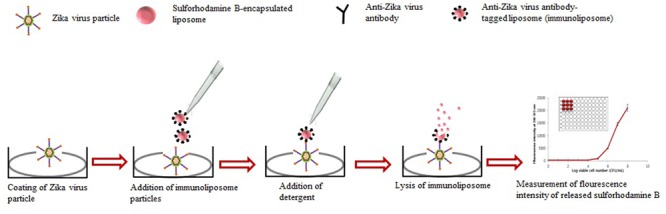
**Strategy for developing liposome immunoassay-based fluorescence immunoassay for the detection of Zika virus**.

**FIGURE 5:**
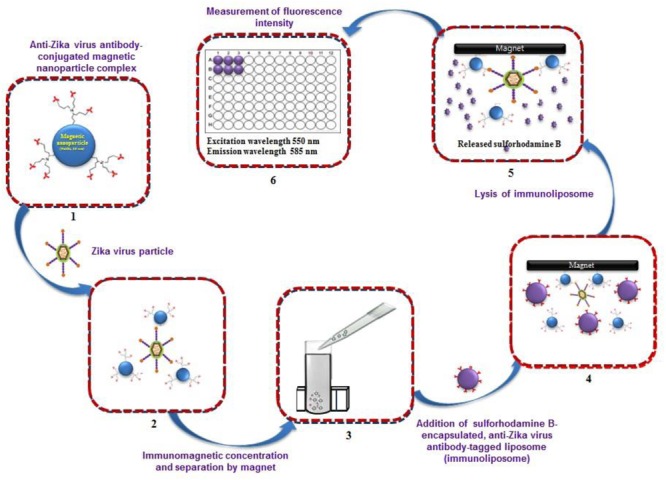
**Strategy for developing immunoliposome and immunomagnetic particle-based sensitive detection of Zika virus. (Retrieved from Shukla et al., 2016)**.

A predicted model of liposome-based immunochromatographic strips for viral detection is summarized in **Figure 3**. Briefly, the assay will involve an antigen capture zone (test line) on a nitrocellulose membrane strip, which is exposed to the target antigen, consisting of a virus particle in a solution containing immunoliposome. Liposomes are encapsulated in sulforhodamine B and tagged with anti-ZIKV antibody specific for the binding of the specific ZIKV particle. In the presence of the ZIKV particle, the immunoliposome and ZIKV particle complex migrates along the test strip via capillary action and subsequently binds to the capture antibody zone of the membrane, producing a purple band. However, some of the immunoliposomes do not bind the ZIKV particles at the test line and continue to migrate and bind to the control line of membrane, resulting in the development of a purple band, showing a positive result for the presence of ZIKV particles (**Figure 3**). In the absence of ZIKV particles in test samples, the immunoliposomes are not able to bind to the specific antigen (ZIKV) and they therefore migrate and bind at control line of the test membrane, leading to the development of a purple line in the control line only, confirming a negative result for ZIKV particle detection (**Figure 3**). In addition, there is another simple liposome-based detection strategy utilizing a liposome immunoassay, in which detection is measured via the lysis of liposome particles (Shukla et al., 2016). Sulforhodamine B is a self-quenching molecule when encapsulated at a high concentration within lipid and phospholipid vesicles to form sulforhodamine B-encapsulated liposomes. Therefore, the integrity of the sulforhodamine B-encapsulated antibody-tagged liposomes with a target virus particle can be determined by measuring the increase in fluorescence after lysis of the immunoliposome particles (**Figure 4**). Furthermore, there might be another strategy for developing a combined assay involving immunoliposomes and immunomagnetic particles for the sensitive detection of ZIKV particles as shown in **Figure 5**. The assay procedure can be divided into the following three steps: immunomagnetic concentration and separation of target ZIKV; reaction of the immunoliposome particles with virus particles attached to immunomagnetic nanoparticles; and fluorescence signal generation, as the principle of the developed assay discussed in our earlier research (Shukla et al., 2016).

**FIGURE 6:**
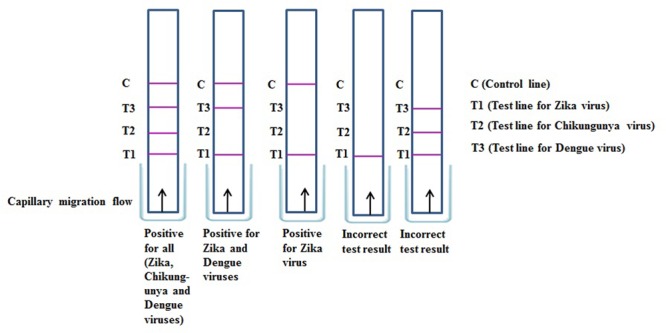
**Result interpretation of immunochromatographic strip assay for simultaneous detection of Zika, Chikungunya, and Dengue viruses**.

Another important possibility is the development of liposome-based multiplexing that would allow simultaneous detection of several similar gene sequences of viral pathogens in a single sample, particularly for ZIKV, DENV, and CHIKV. In addition to ZIKV, DENV, and CHIKV, the choice of pathogens for a multiplexed diagnostic tool should consider clinically consistent presentations. Not all pathogens will be relevant to all settings; nonetheless, the need to differentiate CHIKV, DENV, and ZIKV for both clinical and epidemiologic purposes is of immediate importance and is likely to remain relevant in the future. In multiplexed assays, and particularly those that measure RNA from CHIKV, DENV, and ZIKV, it is important that the analytical sensitivity for ZIKV not be compromised. Possibilities for the interpretation of such multiplexing liposome-based kits, such as those involving immunochromatographic strips, are illustrated in **Figure 6**. To obtain accurate results in multiplexing assays that may be based on liposome-based immunochromatographic strips, the development of a common antibody against ZIKV, DENV, and CHIKV might provide useful results. Multiplex tests should be able to detect the ZIKV antibodies in cases of concurrent infection with both pathogens, and the analytic limit of detection for ZIKV antibodies should be the same for singleplex and multiplex tests. Multiplexing using dual analytes in a single test (e.g., nucleic acid and immunoassay testing) (Chen and Zhu, 2016) may also greatly improve the diagnosis of acute ZIKV infection. For example, a test that could simultaneously detect both ZIKV RNA and anti-ZIKV IgM would cover the entire time period of acute ZIKV infection and might be particularly useful given the limited reliability of patient self-reporting of the onset of fever and other symptoms.

Although few antibody-based ZIKV detection kits are available, we have presented a potentially easy method for the development of pathogen detection kits based on laboratory-produced antibodies and liposomes, which could provide a cheaper alternative for pathogen detection compared with expensive commercial pathogen detection kits. In the near future, studies should attempt to confirm the practical applications of liposome-based virus detection assays in various food samples contaminated with foodborne viruses, including ZIKV.

## Safety Precautions for Laboratories Working on ZIKV

Regarding laboratory safety, ZIKV is classified as a biological safety level 2 pathogenic agent. These pathogens must be handled according to the biosafety guidelines of microbiological and biomedical laboratories, and risk assessment should be performed for each laboratory for any specific procedure used in the laboratory. Because of the major concern regarding congenital microcephaly and ZIKV infection, precautions should be taken by pregnant women while working in the laboratory, and regular ZIKV testing should be maintained in the laboratory. The CDC recommends that each laboratory should perform risk assessment when implementing any new viral methodology in the laboratory as a safety precaution (Centers for Disease Control and Prevention [CDC], 2016a).

## Conclusion

The widespread ZIKV epidemic in the Americas and Asia has led to the urgent need to develop rapid, sensitive, and specific assays for virus detection with regular monitoring of viral infection. Rapid detection of the virus in field-collected specimens can accelerate the application of appropriate mosquito control measures that could prevent transmission and disease among human populations. Early diagnosis of ZIKV infection, supportive care, symptomatic treatment, and referral of children with microcephaly to specialized care are all necessary measures to improve the neuro-development of affected children. The hypothesis and strategies that we have presented herein provide a relevant platform, emphasizing the importance of liposome-based detection methods in ZIKV diagnosis for improving global health. In overall view, the best advantage of these hypothesized assays is easy handling, no requirement of any sophisticated instruments, rapid detection efficacy, and cost effectiveness than other molecular approaches. On the other side, major limiting factor of these hypothesized assays especially for the detection of viral infections including ZIKV, DENV, and others is their antigen-antibody-based detection ability. Identification of major antigenic and protective epitopes of the target virus particles importantly needed to understand the antibody response while developing these detection strategies. In brief, virus particles can easily go for mutation, and antigenic variations by which an infectious virus alters its surface proteins called antigenic structure eventually result in the differentiation of virus strains. These antigenic variations make difficulty in the process of specific antibody production, thus, may affect in the developmental process of these diagnostic assays. In conclusion, the present review summarizes and updates the methods available for the detection of ZIKV in human clinical samples. New insights and interpretations should be obtained by studying the mechanisms of several similar detection strategies that are being developed. Innovative approaches based on liposome particles should be of interest for the detection of ZIKV, particularly in relation to previously developed liposome-based strategies, which may provide new insights for future research and strategies for the development of fast and sensitive assays for ZIKV infections.

## Author Contributions

MK and SS conceived and designed the review theme. S-YH and SHC helped in writing and reviewing the paper.

## Conflict of Interest Statement

The authors declare that the research was conducted in the absence of any commercial or financial relationships that could be construed as a potential conflict of interest.
